# Exome sequencing in families with chronic central serous chorioretinopathy

**DOI:** 10.1002/mgg3.576

**Published:** 2019-02-06

**Authors:** Rosa L. Schellevis, Elon H. C. van Dijk, Myrte B. Breukink, Jan E. E. Keunen, Gijs W. E. Santen, Carel B. Hoyng, Eiko K. de Jong, Camiel J. F. Boon, Anneke I. den Hollander

**Affiliations:** ^1^ Department of Ophthalmology Donders Institute of Brain, Cognition, and Behaviour, Radboud University Medical Center Nijmegen The Netherlands; ^2^ Department of Ophthalmology Leiden University Medical Center Leiden The Netherlands; ^3^ Department of Clinical Genetics Leiden University Medical Center Leiden The Netherlands; ^4^ Department of Ophthalmology Amsterdam University Medical Center, University of Amsterdam Amsterdam The Netherlands; ^5^ Department of Human Genetics Donders Institute of Brain, Cognition, and Behaviour, Radboud University Medical Center Nijmegen The Netherlands

**Keywords:** chronic central serous chorioretinopathy, exome sequencing, families, *PTPRB*, RareIBD

## Abstract

**Background:**

Central serous chorioretinopathy (CSC) is a chorioretinal disease characterized by fluid accumulation between the neuroretina and retinal pigment epithelium with unknown etiology. Family studies have suggested a heritable component for CSC with an autosomal dominant inheritance pattern. Therefore, exome sequencing was performed on familial cCSC to indentify the genetic components contributing to familial cCSC.

**Methods:**

Exome sequencing was performed on 72 individuals of 18 families with CSC. In these families, we determined whether rare genetic variants (minor allele frequency < 1%) were segregated with CSC and also performed familial gene‐burden analysis.

**Results:**

In total, 11 variants segregated in two out of 18 families. One of these variants, c.4145C>T; p.T1382I (rs61758735) in the *PTPRB* gene, was also associated with CSC in a large case–control cohort sequenced previously (*p* = 0.009). Additionally, in 28 genes two or more different heterozygous variants segregated in two or more families, but no gene showed consistent associations in both the family gene‐burden results and gene‐burden analysis in the case–control cohort.

**Conclusion:**

We identified potential candidate genes for familial CSC and managed to exclude Mendelian inheritance of variants in one or a limited number of genes. Instead, familial CSC may be a heterogeneous Mendelian disease caused by variants in many different genes, or alternatively CSC may represent a complex disease to which both environmental factors and genetics contribute.

## 1 INTRODUCTION

In central serous chorioretinopathy (CSC), choroidal congestion, thickening, and hyperpermeability have been suggested to cause leakage through the retinal pigment epithelium (RPE). Subsequently, a neuroretinal detachment occurs due to the accumulation of serous subretinal fluid (Daruich et al., 2015; Gemenetzi, De Salvo, & Lotery, 2010; Liew, Quin, Gillies, & Fraser‐Bell, 2013; Warrow, Hoang, & Freund, 2013; Yannuzzi, 2010). The exact etiology of CSC is still unclear, but male gender and administration of exogenous corticoids have been described to be the most pronounced risk factors for CSC (Carvalho‐Recchia et al., 2002; Haimovici, Koh, Gagnon, Lehrfeld, & Wellik, 2004; Jonas & Kamppeter, 2005). Other risk factors include endogenous hypercortisolism, stress, and pregnancy (Daruich et al., 2015; van Dijk et al., 2016; Liew et al., 2013). Moreover, genetic variants that confer risk or are protective for CSC have been identified by genetic association studies in case–control cohorts (de Jong et al., 2015; Miki et al., 2014; Moschos et al., 2016; Schellevis et al., 2018; Schubert et al., 2014; van Dijk, Schellevis, Bergen et al., 2017).

Although familial occurrence of CSC appears to be rare, several reports on familial CSC and the occurrence of CSC in multiple generations within a single family have been published, pointing to a potential role for genetic factors in familial CSC (Lin, Arrigg, & Kim, 2000; Oosterhuis, 1996; van Dijk, Schellevis, Breukink et al., 2017; Weenink, Borsje, & Oosterhuis, 2001). A Mendelian inheritance of CSC has been proposed previously based on observations that at least two family members proved to have finding characteristics for CSC in 52% of 27 families (Weenink et al., 2001). Moreover, the presence of affected individuals in multiple generations has been described, suggesting an autosomal dominant mode of inheritance of familial CSC (van Dijk, Schellevis, Breukink et al., 2017). Additionally, in 50% of eyes from screened family members of CSC patients, a thickened choroid (pachychoroid) of more than 395 µm was detected, which has been described to be the underlying choroidal abnormality in various diseases that are part of the pachychoroid spectrum (Lehmann, Bousquet, Beydoun, & Behar‐Cohen, 2015). However, thus far no genetic studies on familial CSC have been conducted.

Whole‐exome sequencing has proven to be a powerful tool to identify novel disease‐associated genes and gene variants in many disorders (Gilissen et al., 2010; Gilissen, Hoischen, Brunner, & Veltman, 2012). Exons are presumed to harbor about 85% of disease‐causing mutations, making them a primary target to search for disease‐associated variants in CSC families (Choi et al., 2009). Therefore, we performed exome sequencing on 72 individuals of 18 families in which multiple members were found to have CSC, in order to determine whether Mendelian inheritance of rare genetic variants causes familial CSC.

## 2 MATERIALS AND METHODS

### 2.1 Ethical compliance

Written informed consent for the enrollment was obtained from all subjects. The study adhered to the tenets of the Declaration of Helsinki. Approval of the institutional review boards and the ethics committees was obtained for all centers involved.

### 2.2 Subject selection

In this multicenter study, 72 subjects from 18 families, including patients with CSC and unaffected family members, visited either the Department of Ophthalmology of the Radboud University Medical Center ([Radboudumc] Nijmegen, the Netherlands) or the Leiden University Medical Center ([LUMC] Leiden, the Netherlands). Participants were recruited at the outpatient clinic of the participating hospitals, after the proband had reported a family history of CSC. The majority of the individuals was included in our previously published study on the phenotypic characteristics of familial CSC and was divided in the following groups: “Affected with CSC,” “Suggestive of CSC,” or “Unaffected,” using the criteria described before (van Dijk, Schellevis, Breukink et al., 2017).

Briefly, subjects were categorized as having CSC when serous fluid was detected on an optical coherence tomography scan and when one or more “hot spots” of leakage or diffuse leakage was present in combination with irregular RPE window defects on fluorescein angiography. Patients were excluded if signs of either polypoidal choroidal vasculopathy or age‐related macular degeneration (AMD), or other atypical findings were present. Suggestive CSC was characterized by RPE alterations typical for CSC, but without the presence of either subretinal fluid or 'hot spots' of leakage on fluorescein angiography (van Dijk, Schellevis, Breukink et al., 2017). Unaffected individuals showed no abnormalities on any of the modalities using multimodal imaging.

### 2.3 Exome sequencing

Library preparation of the 52 family members of 12 families collected at the Radboudumc, Nijmegen was performed with the SureSelect^XT^ target enrichment system for Illumina paired‐end multiplex sequencing according to manufacturer's instructions (Version B4, August 2015, Agilent Technologies). Completed libraries were sent to the Department of Genetics of Maastricht University Medical Center+, Maastricht, the Netherlands, where sequencing was performed with eight samples per lane using an Illumina HiSeq2000 with 2*100 bp chemistry, together with a large cohort of 269 sporadic CSC patients (Schellevis et al., 2018 submitted).

The 20 samples of the six families collected at Leiden University Medical Center, Leiden, the Netherlands were sent to GenomeScan BV, Leiden, for sequencing. For these samples, the Agilent SureSelect V5 enrichment kit was used and sequencing was performed with 2*125 bp chemistry on the HiSeq2500.

### 2.4 Variant calling and recalibration

Data of all individuals were processed according to the Genome‐Analysis‐Toolkit (GATK) best practices (v3.8) together with the case–control cohort consisting of 269 sporadic cCSC patients and 1,586 population controls (Schellevis et al., 2018 submitted) to improve variant calling. Briefly, BAM to FastQC extraction was performed with Picard‐tools (v 1.90), duplicate reads were marked with Picard‐tools, and reads were aligned to the reference genome (GRCh37.p5 with alternate haplotypes excluded) using BWA‐MEM (version v.0.7.12), as described before (Schellevis et al., 2018 submitted). Base recalibration was performed and subsequent variant calling was performed with the HaplotypeCaller algorithm. All GVCFs were merged together, and joint genotyping was performed on the entire dataset.

Variant recalibration was performed on the entire dataset with GATK using the recommended settings (McKenna et al., 2010), as described before. Genetic variants located in low complexity regions of the genome were removed (Li, 2014). Multiallelic variants were extracted with VCFtools (v0.1.13) and split using the splitMultiallelic and LeftAlignandTrimVariants option in GATK (v3.8). Variants with a Hardy–Weinberg equilibrium *p* < 1 × 10^−8^ were excluded. Variants from the adult‐onset genes captured in the American College of Medical Genetics and Genomics recommendations for incidental findings (*BRCA1 *[OMIM:113705], *BRCA2 *[OMIM:600185], *MLH1* [OMIM:120436], *MSH2 *[OMIM:609309],* MSH6 *[OMIM:600678], *PMS2 *[OMIM:600259],* MUTYH *[OMIM:604933]) were removed to reduce the risk of secondary findings (Green et al., 2013; Kalia et al., 2017). Finally, family members were extracted from the dataset and only variants with a minor allele count ≥1 in the combined family file were retained. Data were annotated with Tabanno (https://github.com/zhanxw/anno) and Annovar (Yang & Wang, 2015).

### 2.5 Variant filtering

Variants were filtered based on a minor allele frequency (MAF) of ≤1% or ≥99% in the following populations: 1000Genomes_all, 1000Genomes_European, Exac_All, Exac_NFE, Exac_FIN, and esp6500siv2_all. Additionally, variants with MAF ≥1% in the 1586 controls of the sporadic CSC case–control cohort were removed. Variants that were annotated by Annovar to be present in the exonic or splice site regions were retained and synonymous variants were excluded. Remaining variants included: frameshift insertions and deletions (INDELs), nonframeshift INDELs, nonsynonymous variants, stop gain or loss variants, and variants with unknown effects. All variants present in one or more unaffected individuals in any of the families were removed assuming complete penetrance, with the exception of the unaffected individual of *Family 14*, because based on the pedigree structure (Figure S1) reduced penetrance appeared to be present in this family. Only variants with a CADD score above 20, corresponding to the 1% most deleterious variants of the human genome, or with an unknown CADD score in case of INDELs, were retained.

Next, for each family segregation analysis of variants was performed, where variants were retained if they were present in all affected individuals of the family and not present in unaffected individuals. No filtering was performed for individuals with suggestive CSC. Variants that segregated in two or more families or genes that contained multiple variants that segregated in two or more families were retained for further evaluation. Familial gene‐burden associations were calculated with RareIBD for those genes that carried multiple segregating variants in two or more families (Sul et al., 2016). The region encompassing the c.4145C>T; p.T1382I (rs61758735) variant in the *PTPRB *[OMIM:176882] gene was amplified in additional family members of *Family 1* using AmpliTaq DNA polymerase with the following PCR program: 5 min at 95°C, 10 cycles of touchdown starting at 62°C for 45 s and lowering the annealing temperature 0.5°C each cycle, followed by 25 cycles of an annealing temperature of 57°C, all these cycles started with 30 s. at 95°C and ended 45 s at 72°C. The PCR was completed at 5 min, 72°C and the following primers were used forward primer: AGCCTTTGAGCAGCTTTTTC and reverse primer: TGATGCTAGTGCCCCATAAG. The PCR product was analyzed by Sanger sequencing at the core sequencing facility at the Department of Human Genetics of the Radboudumc.

## 3 RESULTS

**Figure 1:**
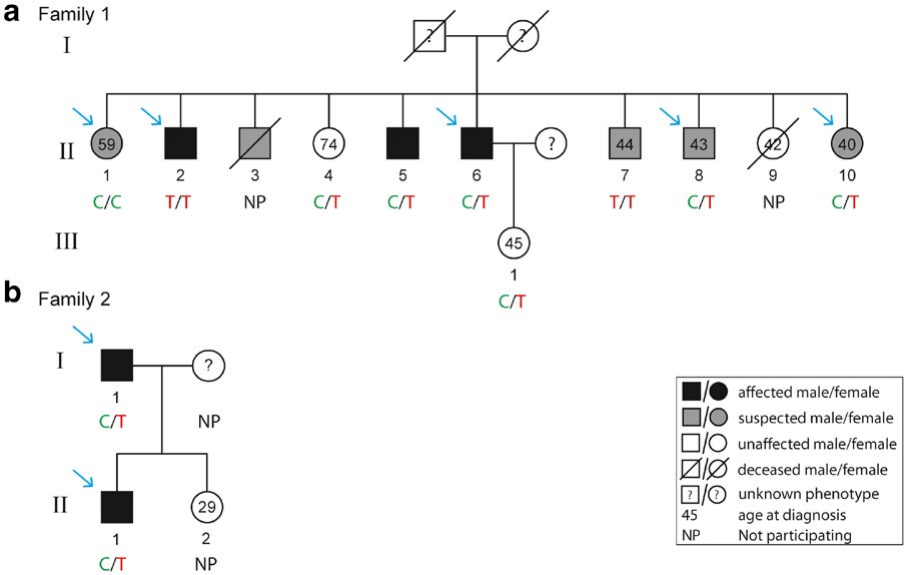
Segregation analysis of c.4145C>T; p.T1382I (rs61758735) in the *PTPRB *gene in *Family 1* (a) and *Family 2* (b). Genotypes for c.4145C>T; p.T1382I (rs61758735) are depicted below each individual in the pedigree. Individuals who were analyzed by exome sequencing are indicated with a blue arrow, while other individuals for whom DNA was available were analyzed by Sanger sequencing. Individual II:2 of family 2 refused to participate in the study, and therefore could not be analyzed

In this exome sequencing study on familial CSC, we included 72 individuals of 18 different families. Out of these 72 individuals, 33 subjects were affected with CSC, 18 had characteristics suggestive of CSC, and 21 were unaffected (Table 1; Figure 1 and Figure S1 for pedigrees). After variant filtering based on MAF (≤1%), CADD score (≥20), absence in unaffected individuals, and protein‐altering effect (frameshift INDELs, nonframeshift INDELs, nonsynonymous, stop gain/loss variants, and variants with unknown effects), the dataset contained 2,806 variants present in 2,368 genes.

**Table 1:** Overview of families analyzed for segregating rare variants using exome sequencing

Family	No. of affected individuals	No. of suggestive individuals	No. of unaffected individuals	No. of segregating variants
Family 1	2	3	–	75
Family 2	2	–	–	69
Family 3	1	3	2	36
Family 4	1	1	4	14
Family 5	1	2	4	3
Family 6	2	2	1	37
Family 7	1	2	3	17
Family 8	3	–	1	17
Family 9	2	–	2	12
Family 10	2	1	–	58
Family 11	2	1	–	79
Family 12	3	–	–	29
Family 13	2	–	–	71
Family 14^a^	2	–	1	37
Family 15	2	–	–	60
Family 16	2	1	1	21
Family 17	1	1	–	124
Family 18	2	1	2	29

a Possibly reduced penetrance in this family.

Segregation analysis was performed for all 18 families, retaining only variants that were present in the affected individuals and absent in unaffected individuals. The average number of segregating variants in each family was 44 and ranged from three to 124 (Table 1). In four families, two segregating heterozygous rare variants in the same gene were observed. *Family 12* carried two variants in *KCNMA1 *[OMIM:600150], *Family 7* in *RBPJL *[OMIM:616104], *Family 8* in *SLC26A10 *[OMIM:NA], and *Family 1* in *SP9 *[OMIM:NA] (Table S1). The entire list of segregating variants for each family is available in Table S2.

Variants that segregated in two or more families were retained for further evaluation. In total, 11 rare variants were found to segregate in two families, of which one variant in the *PTPRB *gene was homozygous in one individual, while the remaining variants in the *SETD2 *[OMIM:612778], *PWP1 *[OMIM:NA], *ABCA9 *[OMIM:612507], *AT2B2 *[OMIM:NA], *ZFAND4 *[OMIM:NA], *MROH5 *[OMIM:NA], *ZAN *[OMIM:602372], *SHISA6 *[OMIM:617327], *DCP1A *[OMIM:607010], and *PPM1E *[OMIM:NA] genes were heterozygous (Table 2). The expression of these genes in the retina and RPE was evaluated using the Eye Integration Database. All genes were expressed in retina and RPE, except for *ZAN* (Figure S2) (Bryan et al., 2018). The single variant association results of these variants were extracted from the sporadic CSC case–control dataset (Schellevis et al., 2018 submitted). Notably, the variant in the *PTPRB *gene (c.4145C>T; p.T1382I, rs61758735) was significantly associated with CSC in the case–control cohort (*p = *0.009, Odds Ratio = 2.83, 95% Confidence Interval = 1.34–5.97). Extended segregation analysis of the *PTPRB* variant in additional available family members of *Family 1* revealed that two individuals carried the variant homozygously (one individual is an affected subject and one is a subject with findings suggestive of CSC), six individuals carried the variant heterozygously (two affected individuals, two individuals with findings suggestive of CSC, and two unaffected individuals), and one individual did not carry the variant (this individual had characteristics suggestive of CSC) (Figure 1a). Since two unaffected individuals (of which one individual was 74 years old at examination) carried the variant heterozygously, complete segregation of this variant with the disease in this family was not observed. For *Family 2*, no additional individuals were available for extended segregation analysis (Figure 1b).

**Table 2:** Rare segregating variants observed in two families

Family	Chr	Position	Rs‐number	Gene	Accession number	Codon change	Protein change	Single variant *p*‐value in case–control cohort^a^	CADD score
Family 7 + 14	3	4,716,897	rs114719990	*SETD2*	NM_014159	c.3229A>G	p.T1077A	0.856	23.6
Family 13 + 17	12	108,105,893	rs11547909	*PWP1*	NM_007062	c.1402G>A	p.E468K	0.801	24
Family 2 + 16	17	67,013,913	rs143651746	*ABCA9*	NM_080283	c.2785T>C	p.F929L	0.167	22.4
Family 1 + 17	3	10,413,597	rs144118750	*ATP2B2*	NM_001683	c.1420G>A	p.V474I	0.092	21.8
Family 9 + 10	10	46,122,195	rs144142701	*ZFAND4*	NM_174890	c.1076T>A	p.L359H	0.592	26.1
Family 2 + 15	8	142,487,895	rs147691391	*MROH5*	NM_207414	c.1346A>T	p.E449V	0.273	24
Family 6 + 17	7	100,365,542	rs183014219	*ZAN*	NM_173059	c.4949C>A	p.T1650K	0.407	24.7
Family 3 + 13	17	11,459,147	rs185956842	*SHISA6*	NM_001173461	c.890C>T	p.P297L	0.241	33
Family 7 + 14	3	53,326,592	rs35988197	*DCP1A*	NM_018403	c.890C>G	p.A297G	0.278	24
Family 1 + 2	12	70,949,014	rs61758735	*PTPRB*	NM_001206971	c.4145C>T	p.T1382I	**0.009**	27.8
Family 1 + 10	17	56,833,502	rs770124556	*PPM1E*	NM_014906	c.144_145insCCCGAA	p.E48delinsEPE	0.375	NA

*p*‐values lower than 0.05 are indicated in bold.

chr, chromosome; NA, not annotated.

a *p*‐value from Schellevis et al., 2018 submitted.

As a next step, genes that contained multiple variants segregating in two or more families were evaluated. In 28 genes, we observed two or more different heterozygous variants that segregated in two or more families, including in two genes known to cause a retinal phenotype (*ABCA4 *[OMIM:601691] and *VCAN *[OMIM:118661]; full list of variants in Table 3). The expression of these genes in the retina and RPE was evaluated using the Eye Integration Database. All genes, with the exception of *AGXT *[OMIM:604285], *LOXHD1 *[OMIM:613072], and *RBPJL*, showed moderate to high expression in the RPE or retina (Figure S3) (Bryan et al., 2018). For all 28 genes, the results of the gene‐based analysis (Burden, SKAT, and SKAT‐O) were extracted from the case–control analysis (Schellevis et al., 2018 submitted). Also, a family gene‐burden analysis was performed with RareIBD including all rare variants found in all families in the 28 genes. Several genes were nominally associated with CSC, but no significant associations were observed in either of the tests after correction for multiple testing of 28 genes (Table 4). Additionally, no genes showed consistent associations in both the case–control cohort and the family cohort.

**Table 3:** Genes with segregating variants in multiple families

Family	Chr	Position	Rs‐number	Gene	Accession number	Codon change	Protein change	Single variant *p*‐value in cases control cohort^a^	CADD score
Family 18	1	94,502,780		*ABCA4*	NM_000350	c.3734G>A	p.S1245N	NA	21.2
Family 14	1	94,508,969	rs61751374	*ABCA4*	NM_000350	c.3113C>T	p.A1038V	0.800	20.5
Family 7	3	183,905,991	rs566108440	*ABCF3*	NM_001351298	c.614G>A	p.R205Q	0.797	26.1
Family 2	3	183,908,940	rs779795407	*ABCF3*	NM_001351298	c.1448C>T	p.P483L	NA	35
Family 17	2	241,815,411	rs140992177	*AGXT*	NM_000030	c.836 T>C	p.I279T	0.201	23.3
Family 1	2	241,810,796	rs121908524	*AGXT*	NM_000030	c.454 T>A	p.F152I	0.588	28.3
Family 7	16	1,394,822	rs148966323	*BAIAP3*	NM_001199096	c.1673C>T	p.T558I	0.496	25.2
Family 2	16	1,394,491	rs114280977	*BAIAP3*	NM_001199096	c.1516G>A	p.D506N	0.052	22
Family 2	8	139,833,569	rs145361557	*COL22A1*	NM_152888	c.1055G>A	p.R352Q	0.823	23.1
Family 12	8	139,838,971	rs72731614	*COL22A1*	NM_152888	c.899G>A	p.R300Q	NA	23.8
Family 18	1	34,015,872	rs149704396	*CSMD2*	NM_052896	c.8390G>A	p.R2797Q	0.079	22.8
Family 14	1	34,066,488	rs755952714	*CSMD2*	NM_001281956	c.6833G>A	p.G2278E	NA	24.9
Family 1	17	1,944,871	rs200625064	*DPH1*	NM_001346576	c.778C>T	p.Q260X	NA	35
Family 2	17	1,943,099	rs80150196	*DPH1*	NM_001346576	c.326C>G	p.P109R	0.429	28.4
Family 4	16	15,733,081	rs150196755	*MARF1/KIAA0430*	NM_001184998	c.10G>A	p.G4R	0.718	28.6
Family 6	16	15,729,650	rs192438053	*MARF1*	NM_001184998	c.694G>A	p.G232R	0.495	25.8
Family 1	6	138,655,606	rs777828045	*ARFGEF3*	NM_020340	c.5624_5626del	p.1875_1876del	0.795	NA
Family 2	6	138,615,130	rs755891726	*ARFGEF3*	NM_020340	c.3369G>T	p.R1123S	0.799	25.3
Family 18	1	109,740,175	rs940837035	*KIAA1324*	NM_001284353	c.1188_1189del	p.I396fs	0.582	NA
Family 4	1	109,734,349	rs41279690	*KIAA1324*	NM_001284353	c.533G>A	p.G178D	0.374	23.3
Family 15	8	28,989,925	rs145324154	*KIF13B*	NM_015254	c.2842G>A	p.A948T	0.573	32
Family 11	8	29,102,864		*KIF13B*	NM_015254	c.148C>G	p.R50G	NA	27.8
Family 13	7	91,871,373	rs34358665	*KRIT1*	NM_001350672	c.77G>A	p.R26Q	0.542	23.6
Family 12	7	91,851,344		*KRIT1*	NM_001013406	c.1291A>G	p.K431E	NA	24.2
Family 17	18	44,089,726	rs571539488	*LOXHD1*	NM_001145473	c.169C>T	p.P57S	NA	25.6
Family 7	18	44,140,215	rs759237437	*LOXHD1*	NM_144612	c.2891_2892insCTCATCAGAGGAGTCCTC	p.S964delinsSSSEESS	0.386	NA
Family 1	14	74,971,538	rs760036288	*LTBP2*	NM_000428	c.4396G>A	p.G1466R	NA	32
Family 15	14	74,967,643		*LTBP2*	NM_000428	c.5410T>G	p.C1804G	NA	27.2
Family 6	17	10,426,647	rs769778269	*MYH2*	NM_001100112	c.5555G>A	p.R1852Q	NA	33
Family 11	17	10,424,643	rs34161789	*MYH2*	NM_001100112	c.5780G>A	p.R1927Q	0.854	35
Family 17	10	95,137,125	rs367618675	*MYOF*	NM_133337	c.2024C>T	p.A675V	NA	22
Family 11	10	95,079,636	rs146626145	*MYOF*	NM_133337	c.5552C>T	p.A1851V	0.248	23.8
Family 3	6	32,180,684	rs150079294	*NOTCH4*	NM_004557	c.2443T>G	p.C815G	0.555	24.5
Family 12	6	32,163,648	rs764118051	*NOTCH4*	NM_004557	c.5578C>A	p.R1860S	NA	34
Family 9	20	47,364,384		*PREX1*	NM_020820	c.253G>A	p.D85N	NA	23.2
Family 11	20	47,282,854	rs149524742	*PREX1*	NM_020820	c.1705G>A	p.V569M	**0.044**	34
Family 6	20	43,945,573	rs199904334	*RBPJL*	NM_001281449	c.1525A>C	p.N509H	NA	25.1
Family 7	20	43,942,164		*RBPJL*	NM_001281448	c.676G>C	p.V226L	NA	27.1
Family 7	20	43,942,170		*RBPJL*	NM_001281448	c.682A>C	p.T228P	NA	26
Family 3	8	145,736,819	rs41555416	*RECQL4*	NM_004260	c.3622C>T	p.R1208C	0.500	23.5
Family 13	8	145,737,142	rs61755067	*RECQL4*	NM_004260	c.3424G>C	p.D1142H	0.585	32
Family 18	3	53,126,560	rs201230044	*RFT1*	NM_052859	c.1283G>A	p.S428N	NA	22
Family 12	3	53,126,512	rs147740901	*RFT1*	NM_052859	c.1331C>T	p.T444M	0.588	22.7
Family 17	9	135,173,569		*SETX*	NM_001351527	c.5679G>A	p.M1893I	NA	25.1
Family 12	9	135,210,039	rs527394446	*SETX*	NM_001351527	c.794A>G	p.D265G	0.659	28.5
Family 17	16	89,965,023	rs144328773	*TCF25*	NM_014972	c.1081C>A	p.R361S	0.673	34
Family 18	16	89,972,604	rs137901241	*TCF25*	NM_014972	c.1631G>A	p.R544Q	0.811	28.4
Family 17	2	179,554,624	rs202234172	*TTN*	NM_133378	c.28031–1G>A	—	0.360	26.1
Family 15	2	179,628,969	rs139504522	*TTN*	NM_003319	c.9911C>T	p.P3304L	NA	23.6
Family 13	2	179,396,568	rs201218828	*TTN*	NM_003319	c.77579A>C	p.E25860A	0.241	22.1
Family 11	2	179,411,137		*TTN*	NM_003319	c.67726G>A	p.G22576S	NA	23.3
Family 14	9	132,636,952	rs142714756	*USP20*	NM_001008563	c.1838G>A	p.R613H	NA	32
Family 12	9	132,630,423	rs148425010	*USP20*	NM_001008563	c.830G>A	p.S277N	0.379	23.2
Family 10	5	82,835,589	rs146630369	*VCAN*	NM_001164097	c.3806 T>C	p.L1269P	0.796	24.2
Family 11	5	82,850,808	rs768896921	*VCAN*	NM_001126336	c.1463A>G	p.N488S	NA	28.9
Family 13	14	75,245,347		*YLPM1*	NM_019589	c.1072_1074del	p.358_358del	NA	NA
Family 11	14	75,265,490		*YLPM1*	NM_019589	c.3490C>G	p.R1164G	NA	25.5
Family 17	3	102,183,076	rs375032047	*ZPLD1*	NM_175056	c.790C>T	p.R264X	0.603	48
Family 15	3	102,175,036		*ZPLD1*	NM_175056	c.376–1G>A	—	NA	25.7

a *p*‐values lower than 0.05 are indicated in bold; *p*‐value from Schellevis et al., 2018 submitted; chr, chromosome; NA, not annotated.

**Table 4:** Gene‐based analysis results of the genes with multiple segregating variants in two or more families

Gene	Case–control analysis^a^			Family analysis
	Burden	SKAT	SKAT‐O	RareIBD
*ABCA4*	0.074	0.295	0.129	0.051
*ABCF3*	0.386	1.000	0.523	**0.047**
*AGXT*	0.374	0.367	0.517	0.301
*ARFGEF3*	0.126	0.166	0.198	0.085
*BAIAP3*	0.076	0.663	0.132	0.143
*COL22A1*	0.564	0.506	0.684	**0.020**
*CSMD2*	0.460	0.266	0.471	0.328
*DPH1*	**0.039**	0.220	0.061	0.086
*KIAA1324*	0.074	**0.035**	0.051	0.097
*KIF13B*	0.053	**0.026**	**0.044**	0.288
*KRIT1*	0.345	0.821	0.446	**0.021**
*LOXHD1*	0.567	0.960	0.761	**0.049**
*LTBP2*	0.378	0.827	0.553	0.177
*MARF1*	0.710	1.000	0.878	0.094
*MYH2*	0.858	0.452	0.655	**0.037**
*MYOF*	**0.016**	0.128	**0.025**	0.144
*NOTCH4*	0.544	0.939	0.740	**0.018**
*PREX1*	**0.044**	0.165	0.073	0.061
*RBPJL*	0.131	0.262	0.195	**0.014**
*RECQL4*	0.234	0.538	0.391	0.144
*RFT1*	0.073	0.341	0.130	**0.019**
*SETX*	0.587	0.494	0.667	0.169
*TCF25*	0.782	0.594	0.829	0.191
*TTN*	0.577	0.435	0.596	0.072
*USP20*	0.226	0.948	0.354	0.215
*VCAN*	0.271	1.000	0.414	0.144
*YLPM1*	0.524	0.871	0.715	**0.047**
*ZPLD1*	0.237	1.000	0.373	0.110

a *p*‐values lower than 0.05 are indicated in bold; Burden, SKAT, and SKAT‐O results were obtained from a recent case–control study performed with 263 sporadic CSC patients and 1,352 population controls (Schellevis et al., 2018 submitted).

## 4 DISCUSSION

In this exome sequencing study on familial CSC, we included 72 individuals of 18 different families and focused on rare genetic variants that segregated with the disease in these families. We observed 11 variants that segregated in two families, of which one was also associated with CSC in a recent case–control study (Schellevis et al., 2018 submitted). In addition, in 28 genes two different variants were found to segregate in two families, and 25 of these genes showed expression in the retina or RPE according to the Eye Integration Database.

For AMD, a well‐studied multifactorial eye disease with phenotypic overlap with CSC, exome sequencing studies in families have been successful in identifying rare variants that fully or partially segregate with the disease (Geerlings et al., 2017; Hoffman et al., 2014; Pras et al., 2015; Saksens et al., 2016; Wagner et al., 2016; Yu et al., 2014). Most variants in these AMD families were identified in genes of the complement system, in which common and rare variants were previously identified to be associated with AMD in case–control cohorts (Fritsche et al., 2016). So far, the only genetic association that has been consistently been replicated in CSC was identified for common variants in the *complement factor H* (*CFH *[OMIM:134370]) gene (de Jong et al., 2015; Miki et al., 2014; Moschos et al., 2016; Schellevis et al., 2018). However, in this study, we did not observe any segregating rare variants in *CFH *in CSC families*.*

In this first unbiased exome sequencing study in a large cohort of families with CSC, we did not identify either a single variant or multiple variants in a single gene that segregated with the CSC phenotype. This excludes that familial CSC is a Mendelian disease caused by mutations in a single gene. Analysis of exome sequencing data identified numerous variants that segregate with the disease in each individual family. However, with this study setting, it is impossible to identify which of these variants might have an effect on the phenotype. Therefore, we focused on variants that segregated with CSC in at least two families, and on genes that carried multiple variants that segregated with CSC in at least two families.

In total, 11 segregating variants were observed in two families, of which the c.4145C>T; p.T1382I (rs61758735) variant in the *PTPRB* gene showed an association in the sporadic CSC case–control cohort (Schellevis et al., 2018 submitted). However, extended segregation analysis in additional family members excluded complete segregation of the *PTPRB* variant with the disease in one of two families. This is in line with results obtained in AMD families, in which rare variants did not always completely segregate with the disease (Duvvari et al., 2016; Geerlings et al., 2017; Hoffman et al., 2014; Saksens et al., 2016). Nevertheless, these variants are likely to contribute to the disease in these families, since several rare variants that are significantly associated with AMD in case–control studies often also show partial segregation in families (Geerlings et al., 2017; Saksens et al., 2016).

The *PTPRB* gene encodes the vascular endothelial protein tyrosine phosphatase (VEPTP) protein. Vascular endothelial protein tyrosine phosphatase is an important modulator of vascular endothelium morphogenesis and is involved in promoting angiogenesis and in regulating endothelial barrier functions by interacting with cadherin (Baumer et al., 2006; Nottebaum et al., 2008). Furthermore, intra‐ocular injections of anti‐VEPTP have been found to suppress neovascularization in mice (Shen et al., 2014). As it has been hypothesized that choroidal hyperpermeability and dysfunction is the most important underlying problem in CSC (Daruich et al., 2015), the *PTPRB* gene is an interesting candidate gene for CSC and should be investigated in future studies. Variants in the *PTPRB *gene could potentially predispose individuals to an impaired vascular network resulting in and leakage and occurrence of CSC. The remaining 11 variants were very rare or even absent in the case–control cohort, and we can neither rule out nor confirm their possible role in the CSC disease mechanism at this time.

In 28 genes, multiple different segregating variants were observed in at least two families. For these genes, gene‐based associations in the case–control cohort (using SKAT, SKAT‐O, and Burden test) and gene‐based associations in the CSC families (using RareIBD) were evaluated. Five genes were nominally associated in the gene‐burden analyses of the case–control cohort and nine genes were nominally associated in the family dataset. However, none of the genes showed consistent associations in both the gene‐burden analysis in the case–control cohort and in the family gene‐burden analysis. Of the five genes nominally associated in the case–control cohort, the *DPH1 *[OMIM:603527], *KIAA1324 *[OMIM:611298], and *PREX1 *[OMIM:606905] genes showed a trend toward association in the family dataset and might be interesting genes for replication in a larger CSC cohorts.

In summary, we aimed to identify rare variants associated with familial CSC. In each family, many variants segregated with the disease, but only few were found to segregate in at least two families. One of these variants was also associated with CSC in a recent case–control cohort (Schellevis et al., 2018 submitted), and this gene, *PTPRB*, has a function that could be of importance in the etiology of CSC. Therefore, *PTPRB *might be an interesting candidate gene for future studies on CSC. Future analyses should include additional families with more individuals to increase the chance of finding segregating variants in multiple families, and to increase the power of the RareIBD analysis. However, this may be challenging due to the relatively rare occurrence of familial CSC. Additionally, future studies may focus on the potential role of genetic variants in noncoding genetic regions, such as introns and promoter regions or large structural alterations that cannot be detected with exome sequencing, such as copy number variations.

In general, in familial CSC, a Mendelian inheritance pattern of variants in one or a limited number of genes can be excluded based on this study. Instead, familial CSC may be a heterogeneous Mendelian disease caused by variants in many different genes, or alternatively CSC may represent a complex disease to which both genetic and environmental factors contribute.

## CONFLICT OF INTEREST

None declared.
