# Supplementary material for: Exome sequencing in families with chronic central serous chorioretinopathy
Source: Mol Genet Genomic Med. 2019 Feb 6;7(4):e00576. doi: 10.1002/mgg3.576 (PMC6465660; doi:10.1002/mgg3.576)
Supplement: Supplementary file 1 [file MGG3-7-na-s001.pdf]

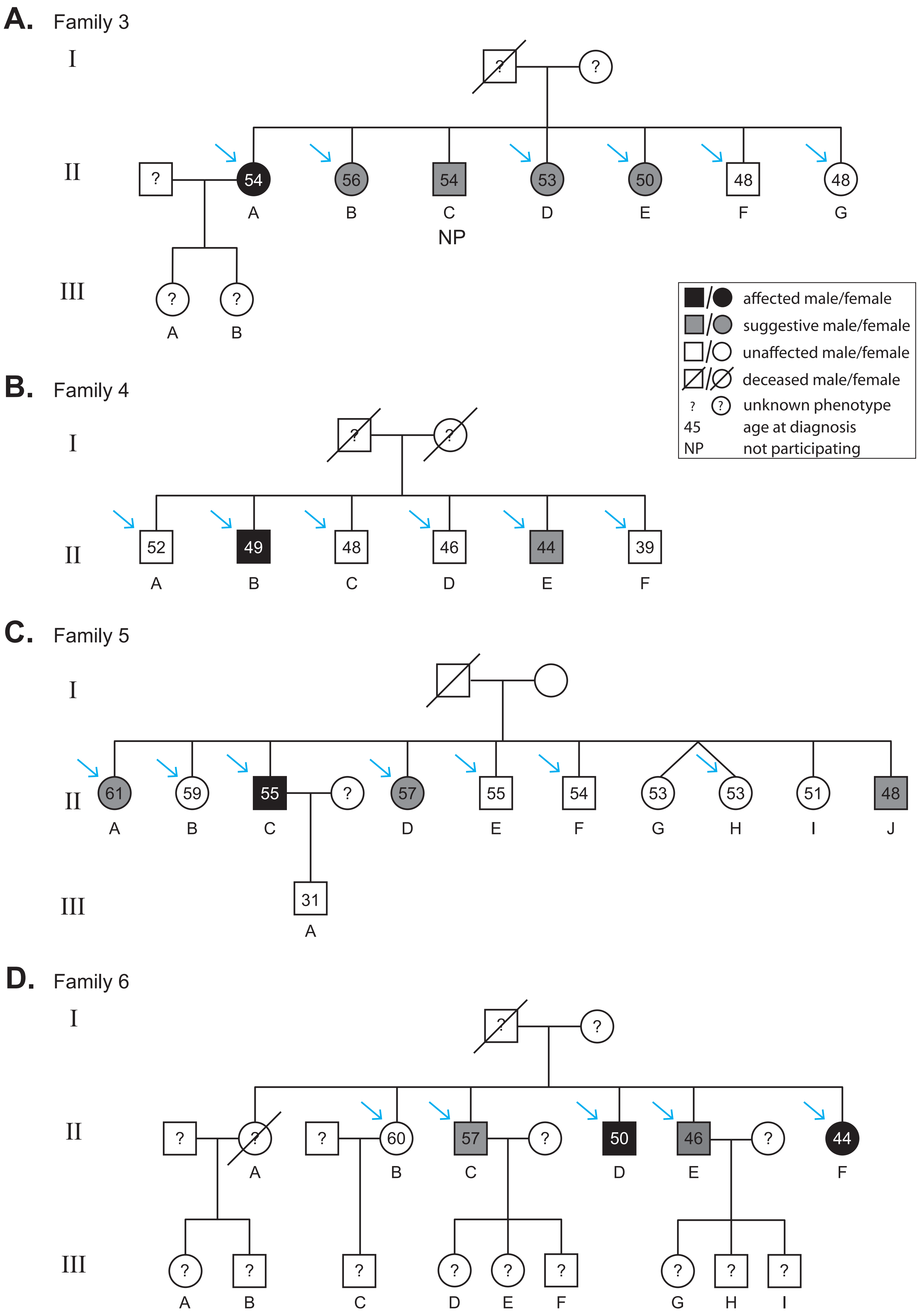

A. Family 3
I
?
?
II
?
54
A
56
B
54
C
NP
53
D
50
E
48
F
48
G
III
?
A
?
B
affected male/female
suggestive male/female
unaffected male/female
deceased male/female
? unknown phenotype
45 age at diagnosis
NP not participating
B. Family 4
I
?
?
II
52
A
49
B
48
C
46
D
44
E
39
F
C. Family 5
I
II
61
A
59
B
55
C
?
57
D
55
E
54
F
53
G
53
H
51
I
48
J
III
31
A
D. Family 6
I
?
?
II
?
?
A
?
60
B
57
C
?
50
D
46
E
?
44
F
III
?
A
?
B
?
C
?
D
?
E
?
F
?
G
?
H
?
I

**E.** Family 7

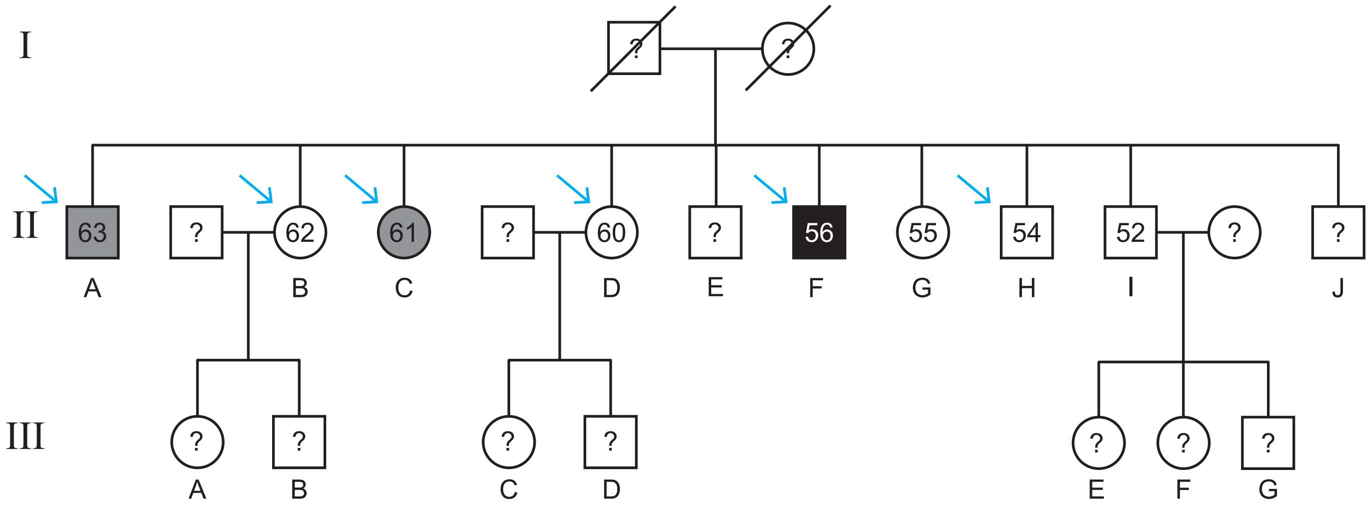


**F.** Family 8

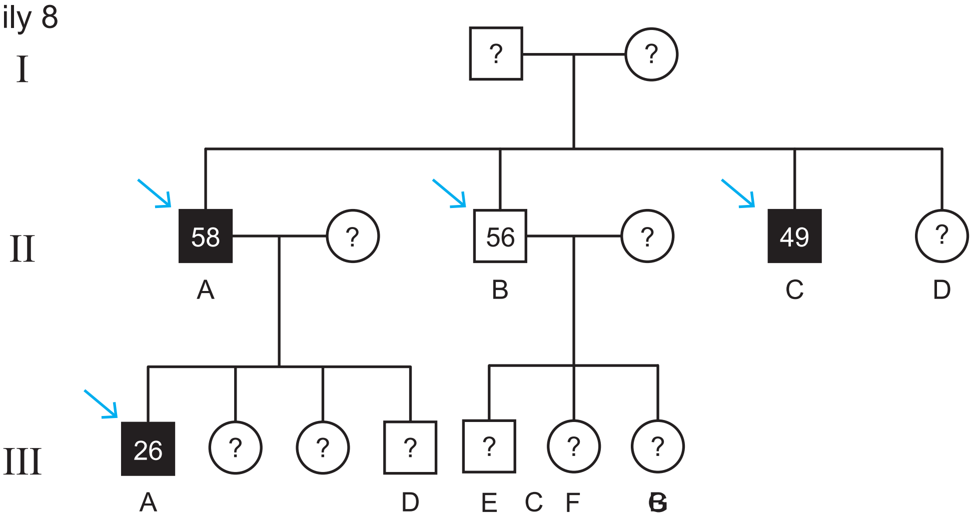


**G.** Family 9

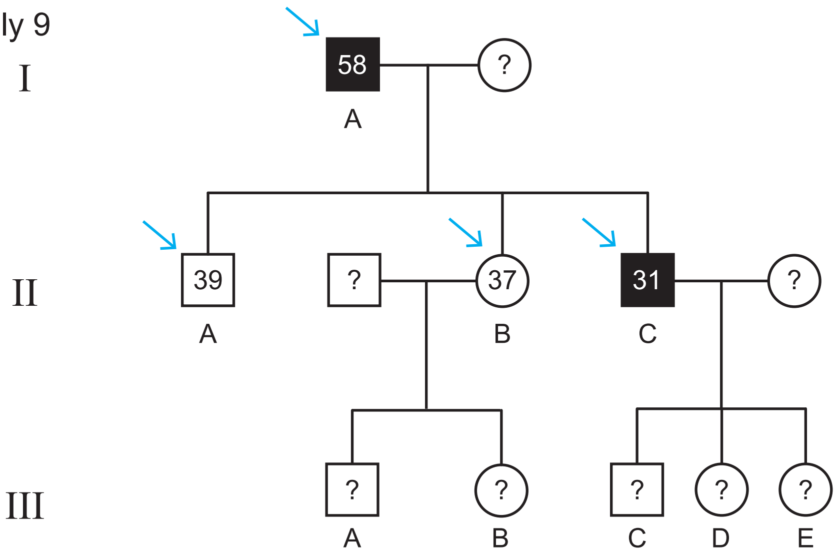

**H.** Family 10

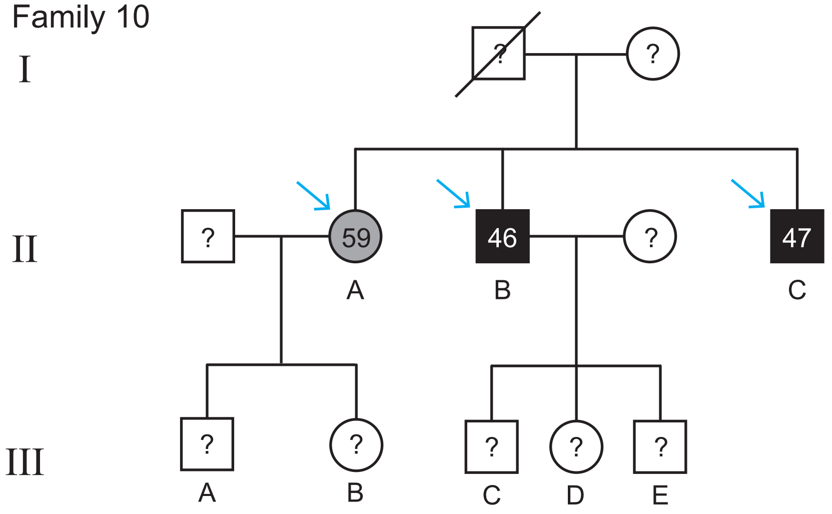


**I.** Family 11

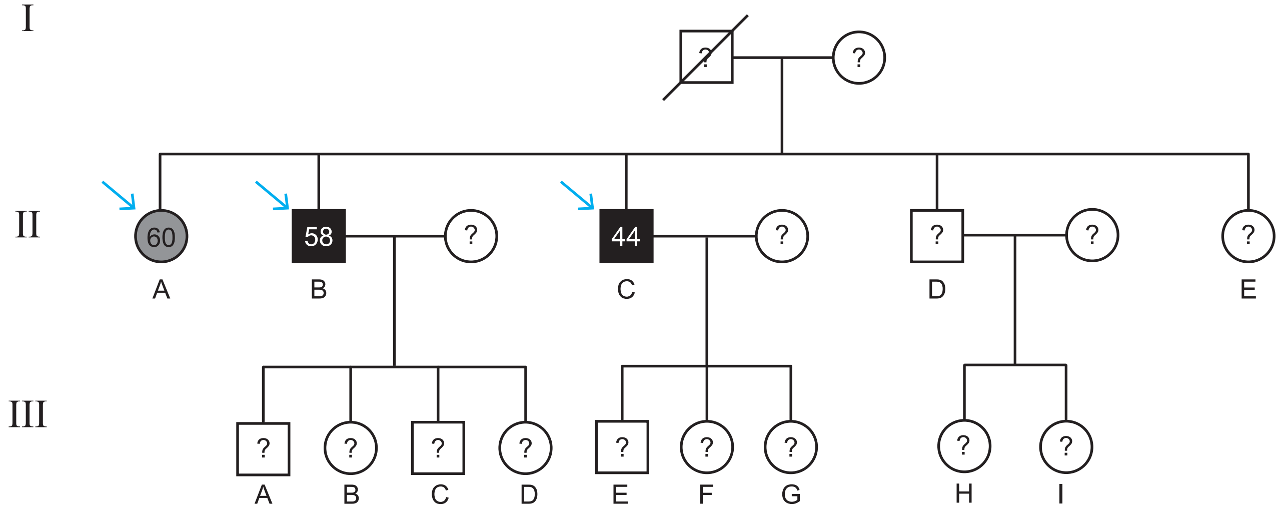


**J.** Family 12

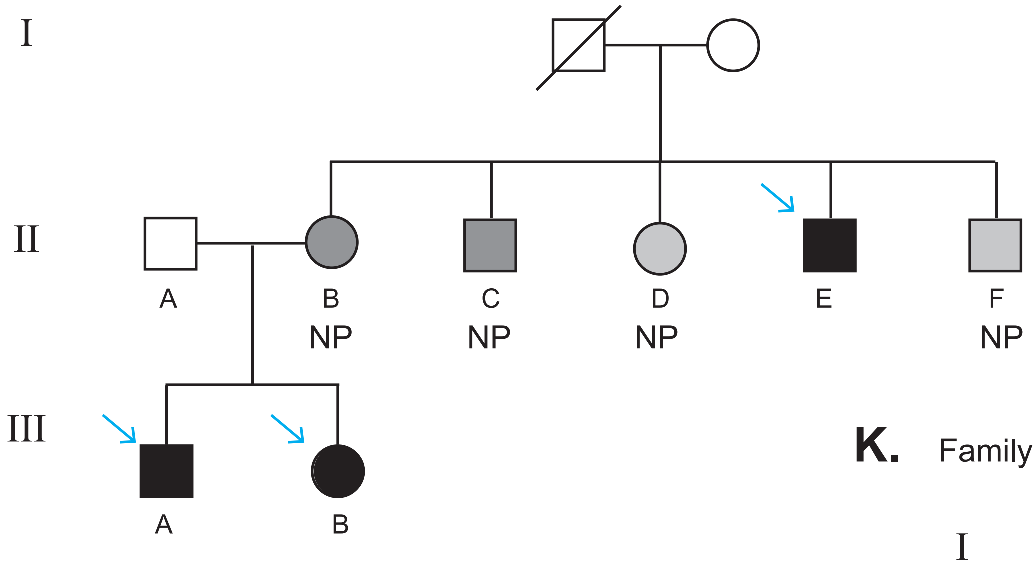


**K.** Family 13

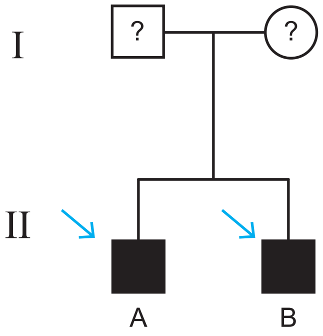

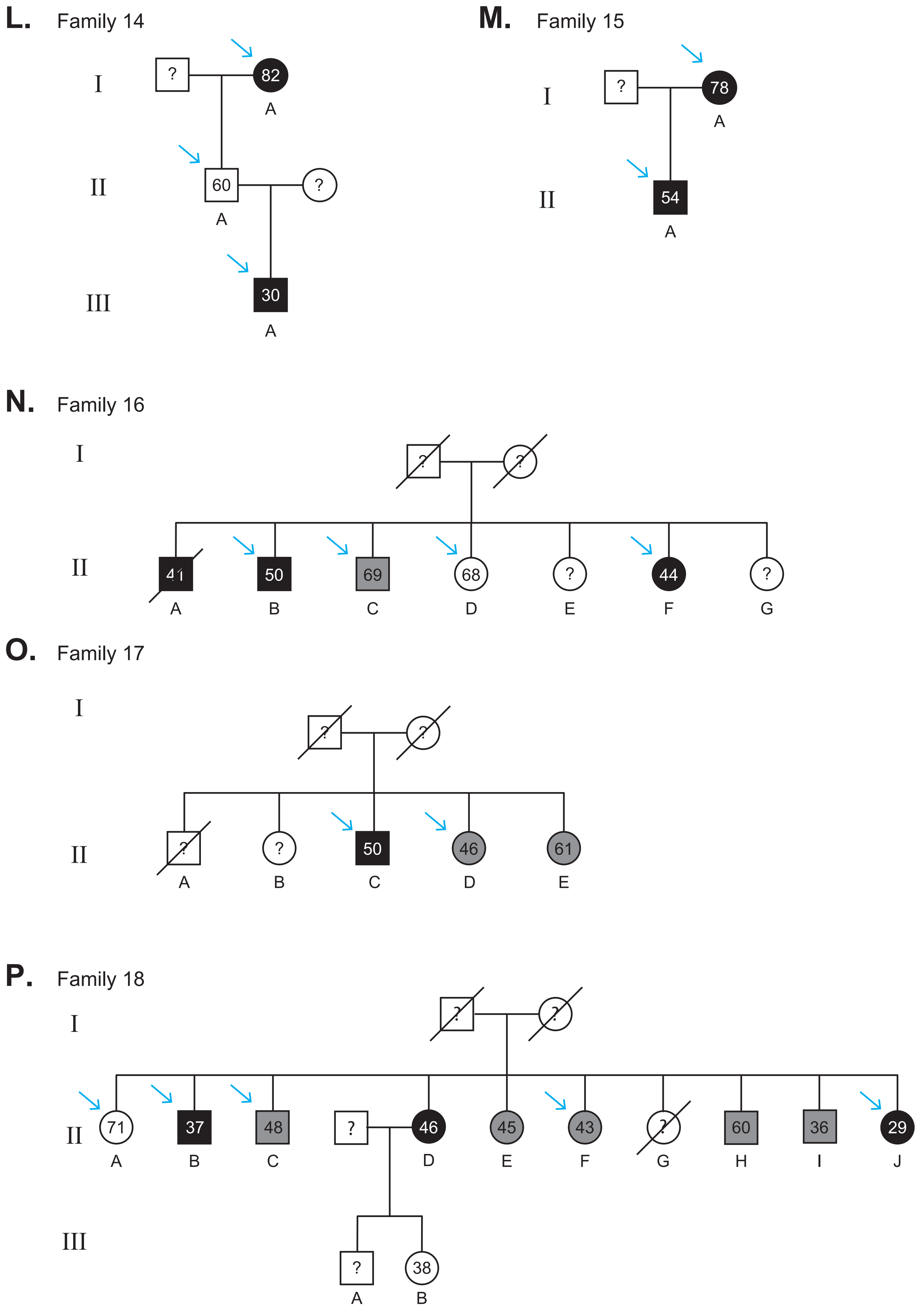

L. Family 14
I
?
82
A
II
60
A
?
III
30
A
M. Family 15
I
?
78
A
II
54
A
N. Family 16
I
?
?
II
41
A
50
B
69
C
68
D
?
E
44
F
?
G
O. Family 17
I
?
?
II
?
A
?
B
50
C
46
D
61
E
P. Family 18
I
?
?
II
71
A
37
B
48
C
?
46
D
45
E
43
F
?
G
60
H
36
I
29
J
III
?
A
38
B
