# Supplementary figures and images for: Exome sequencing in families with chronic central serous chorioretinopathy
Source: Mol Genet Genomic Med. 2019 Feb 6;7(4):e00576. doi: 10.1002/mgg3.576 (PMC6465660; doi:10.1002/mgg3.576)

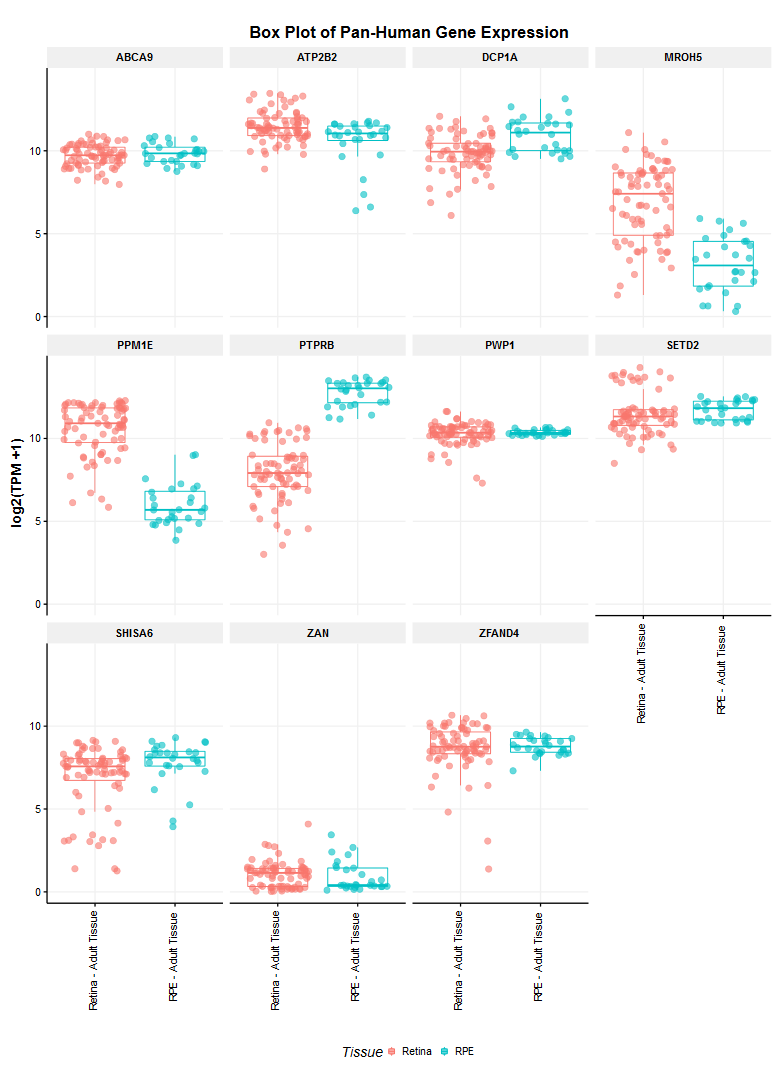

Supplement: Supplementary file 2 [file MGG3-7-na-s002.png]

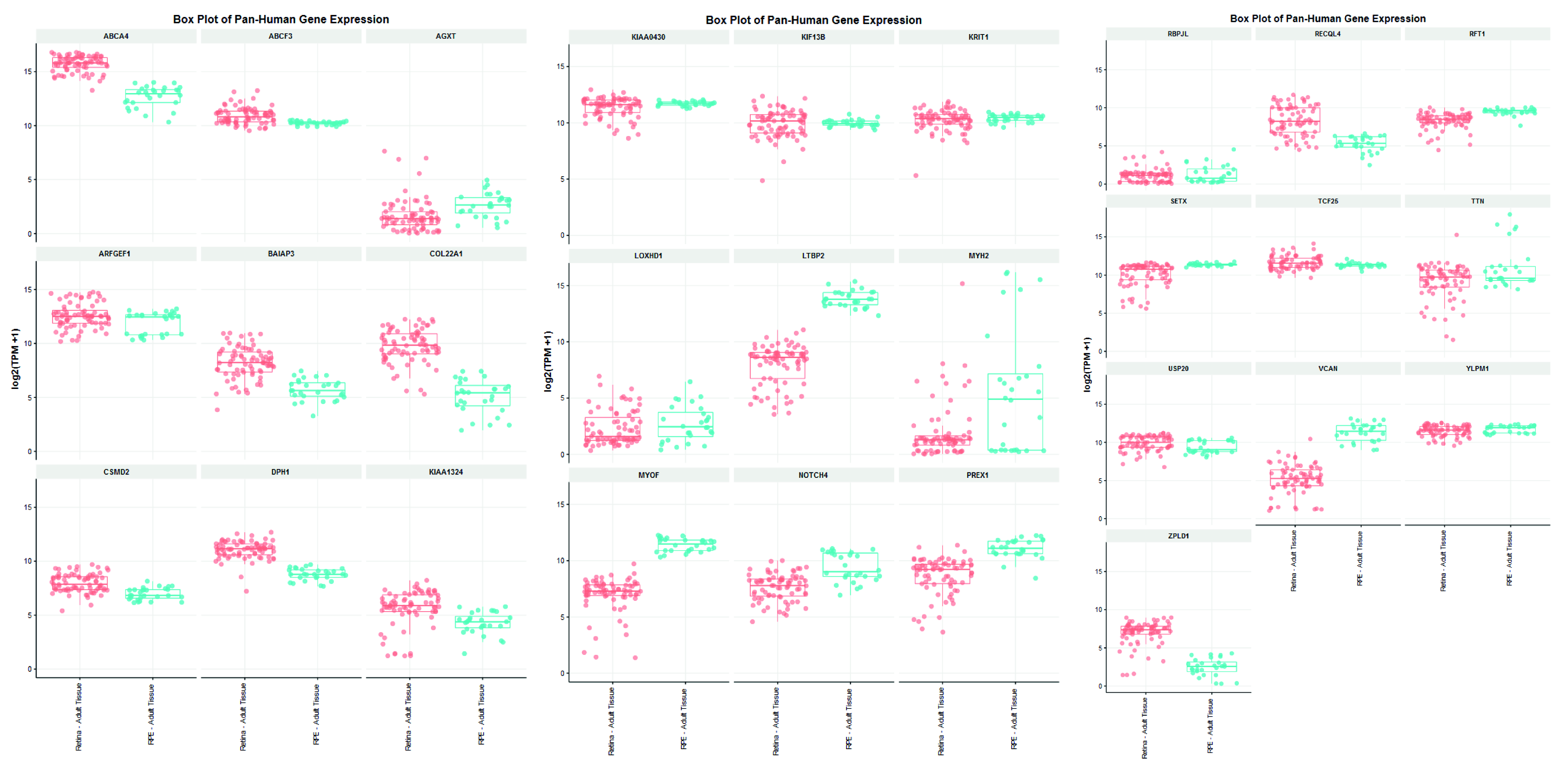

Supplement: Supplementary file 3 [file MGG3-7-na-s003.tif]
