# Supplementary material for: Exome sequencing in families with chronic central serous chorioretinopathy
Source: Mol Genet Genomic Med. 2019 Feb 6;7(4):e00576. doi: 10.1002/mgg3.576 (PMC6465660; doi:10.1002/mgg3.576)
Supplement: Supplementary file 6 [file MGG3-7-na-s006.docx]

**Supplementary Figure 1. Pedigree structures of included cCSC families.**

**Supplementary Figure 2. Expression of the 11 genes carrying segregating variants in two families in adult RPE and retina (Eye Integration Database).**

**Supplementary Figure 3. Expression of the 28 genes carrying multiple different segregating variants in more than two families in adult RPE and retina (Eye Integration Database).**
